# Clinical Utility of Liquid Biopsy for the Early Diagnosis of EGFR-Mutant Advanced Lung Cancer Patients in a Real-Life Setting (CLEAR Study)

**DOI:** 10.3390/curroncol32020057

**Published:** 2025-01-21

**Authors:** Ramy Samaha, Rola El Sayed, Raafat Alameddine, Marie Florescu, Mustapha Tehfe, Bertrand Routy, Arielle Elkrief, Wiam Belkaid, Antoine Desilets, Xiaoduan Weng, Rami Nassabein, Félix Blanc-Durand, Gurvinder Kenth, Goulnar Kasymjanova, Jason Agulnik, Normand Blais

**Affiliations:** 1 Hematology/Oncology, Centre Hospitalier de l’Université de Montréal, Montreal, QC H2X 3E4, Canada; ramy.samaha.med@ssss.gouv.qc.ca (R.S.); rola.el.sayed.med@ssss.gouv.qc.ca (R.E.S.); raafat.alameddine@gmail.com (R.A.); marie.florescu.med@ssss.gouv.qc.ca (M.F.); mustapha.tehfe.med@ssss.gouv.qc.ca (M.T.); bertrand.routy.med@ssss.gouv.qc.ca (B.R.); arielle.elkrief.med@ssss.gouv.qc.ca (A.E.); wiam.belkaid.chum@ssss.gouv.qc.ca (W.B.); antoine.desilets.med@ssss.gouv.qc.ca (A.D.); xiaoduan.weng.chum@ssss.gouv.qc.ca (X.W.); rami.nassabein.med@ssss.gouv.qc.ca (R.N.); felix.blanc-durand@gustaveroussy.fr (F.B.-D.); 2 Axe Cancer, CRCHUM—Centre de Recherche du CHUM, Montreal, QC H2X 0A9, Canada; 3 Oncology, AstraZeneca Canada, Inc., Mississauga, ON L4Y 1M4, Canada; gurvinder.kenth@astrazeneca.com; 4 The Anne and Peter Brojde Lung Cancer Centre, Jewish General Hospital, Montreal, QC H3T 1E2, Canada; gkasymja@jgh.mcgill.ca; 5 Pulmonary and Medical Oncology, Jewish General Hospital, Montreal, QC H3T 1E2, Canada; jason.agulnik.med@ssss.gouv.qc.ca

**Keywords:** lung cancer, non-small cell lung cancer (NSCLC), liquid biopsy (LBx), circulating tumor DNA (ctDNA), next-generation sequencing (NGS), molecular diagnostics, personalized medicine

## Abstract

**Background:** Lung cancer remains the leading cause of cancer mortality globally with EGFR mutations representing a significant driver in advanced non-small cell lung cancer (aNSCLC). The timely detection of these mutations is critical for initiating targeted therapy, yet tissue biopsy limitations often delay treatment. **Methods:** This multicenter prospective study evaluated the clinical utility of liquid biopsy (LBx) in real-life settings for the early diagnosis of EGFR mutations in patients with suspected aNSCLC. Circulating tumor DNA (ctDNA) was analyzed using the Cobas EGFR Mutation Test and compared to tissue-based next-generation sequencing (NGS). **Results:** Among 366 aNSCLC patients tested, LBx demonstrated a significantly shorter median turnaround time (TAT) of 3 days compared to 26 days for tissue NGS (*p* < 0.001) with 100% specificity and 65% sensitivity for EGFR mutation detection. LBx identified actionable EGFR mutations in cases where tissue biopsy was insufficient or unavailable, enabling 43.7% of patients to commence targeted therapy based on ctDNA results prior to biopsy confirmation. **Conclusions:** These findings highlight the potential of LBx to reduce diagnostic delays and improve access to personalized therapies in a real-world setting. Integrating LBx into routine diagnostic workflows may address current gaps in molecular testing, ensuring timely and precise treatment for aNSCLC patients.

## 1. Introduction

Lung cancer remains a pressing public health challenge, representing the most prevalent type of cancer globally. Its incidence and associated mortality rates underscore the urgent need for comprehensive strategies to address this serious malignancy [1]. Today, the management of aNSCLC has transitioned into an era of personalized and targeted therapy guided by molecular subtyping. The IPASS trial was the first study that showed the importance of molecular testing in aNSCLC more than 15 years ago [2]. Since then, many genomic alterations, for which drugs are approved in the clinical setting, have been identified. Molecular testing to identify these alterations has become standard-of-care according to the European Society of Medical Oncology (ESMO) and the American Society of Clinical Oncology (ASCO) guidelines with important implications on treatment selection and outcome [3,4]. Epidermal growth factor receptor (*EGFR*) mutations are the most common driver mutations found in patients with lung adenocarcinomas and occasionally found in squamous cell carcinomas. Prevalence is estimated at 49.1% in east Asia, 12.8% in Europe and 15.2% in Canada [5,6]. EGFR tyrosine kinase inhibitors (TKIs) are superior to chemotherapy in *EGFR*-mutant (EGFRm) non-small cell lung cancer (NSCLC) and are associated with a relatively rapid time to response [7,8].

Historically tissue biopsy was used to detect genomic alterations before starting treatment in aNSCLC, although this is not always possible in a real-world setting due to the inability to acquire tissue, sequencing failure or lack of resources [9,10]. Next-generation sequencing (NGS) testing and single-gene testing are the two primary testing strategies currently available in Canada. Targeted NGS is often regarded as the preferred technique due to its ability to analyze a broader panel of genes, enhanced cost-effectiveness, and reduced need for repeat testing ultimately leading to faster treatment initiation [11,12,13,14]. However, the turnaround time (TAT) from reception of biopsy to start of treatment remains 5.1 weeks compared to 9.2 weeks in single-gene strategies [15]. In 2018, a Canadian investigator reviewed the TAT for *EGFR* molecular reporting between biopsy procedure and issuance of molecular pathology report sign out. The median TAT was 23 days and 34 days in two large referral centers in Montreal [16].

These delays can negatively impact treatment, leading the National Comprehensive Cancer Network (NCCN) guidelines to recommend initiating empiric upfront treatment while awaiting testing results [17]. However, a study by Scott et al. demonstrated that initiating treatment before genomic test results are available can adversely affect time to next treatment and overall survival (OS) in patients with an actionable molecular alteration [18]. Consequently, promptly and adequately selecting the optimal first-line treatment is imperative. In this context, liquid biopsy (LBx) presents itself as an appealing complementary option.

The term “liquid biopsy” was first used by Pantel and Alix-Panabières to refer to the practice of obtaining diagnostic information by means of blood sampling rather than traditional tissue biopsies [19]. For instance, circulating tumor DNA (ctDNA) assays through NGS have been developed as a non-invasive tool for the monitoring of response to anticancer therapy, detecting minimal residual disease (MRD) in early-stage cancer, screening for cancer, and aiding in treatment selection for patients with advanced cancer [20,21]. LBx involves sequencing ctDNA to detect actionable biomarkers and guide targeted therapy for aNSCLC. Specifically, it analyzes circulating cell-free DNA derived from tumor cell apoptosis and necrosis and obtained from a blood sample. This extracted ctDNA can then be amplified, analyzed, and assessed for mutations [22,23]. In the context of NSCLC, ctDNA LBx is increasingly utilized for genotyping, either in combination with or as a substitute for tissue biopsies, particularly when the latter is inadequate for NGS or technically challenging. Broad targeted NGS panels have been developed to detect genomic alteration with high accuracy such as MSK-IMPACT^®^ in tissue and MSK-ACCESS^®^ in plasma [24].

There are many emerging applications for LBx in NSCLC related to cancer screening, diagnosis and MRD detection. Among FDA-approved tests to identify actionable mutations in LBx, *Idylla^TM^ ctEGFR Mutation Assay* and *Cobas^®^ EGFR Mutation Test v2* both employ real-time polymerase chain reaction (PCR) techniques and are associated with low TAT [25]. The aim of our study was to demonstrate that LBx has the potential to significantly reduce TAT compared to tissue molecular diagnosis without compromise on sensitivity and specificity, expediting the initiation of adequate treatment for individuals diagnosed with aNSCLC.

## 2. Methods

### 2.1. Study Design

CLEAR was a multicenter, prospective, non-randomized study that enrolled patients with radiological suspicion of lung cancer and no prior biopsy or cytology confirming a diagnosis of aNSCLC. The study was conducted at Centre hospitalier de l’Université de Montréal (CHUM), with the participation of other hospitals in Quebec, including the Jewish General Hospital, Maisonneuve-Rosemont Hospital, Sacré-Cœur-de-Montréal Hospital, Cité-de-la-Santé Hospital, Pierre-Boucher Hospital, Anna-Laberge Hospital, St-Jerome Hospital, Notre-Dame Hospital, Saint-Eustache Hospital, Saint-Hyacinthe Hospital, Charles-Lemoyne Hospital, and Hôtel-Dieu-de-Sorel Hospital.

### 2.2. Patient Recruitment and Biopsy Sampling

Patients suspected to have primary lung cancer were identified through referrals by oncologists, thoracic surgeons, pulmonologists, and radiation oncologists from the different participating centers. Eligible patients included men and women over 18 years of age with suspected aNSCLC on imaging. Patients with histologically proven lung cancer or other malignancies and those already receiving systemic therapy were excluded. Upon screening, a study coordinator scheduled meetings with the patients to discuss the study and obtain informed consent. During these meetings, detailed interviews were conducted to gather relevant medical history and demographic characteristics such as sex, age, Eastern Cooperative Oncology Group (ECOG) performance status, smoking history, and prior history of cancer. Whenever feasible, retrospective data curation from chart review was also performed to assess anti-EGFR TKI treatment initiation.

Following the consent process, blood samples were collected via phlebotomy using Streck tubes. DNA extraction was performed using the Cobas *EGFR* mutation test kit c.2 with PCR conducted on a Cobas system analyzer (Roche Diagnostics, Indianapolis, IN, USA). Finally, the referring physician was promptly informed of the test result to guide further clinical management. TAT from LBx blood sampling to *EGFR* test result reporting was recorded.

Patients underwent histologic sampling as per standard of practice. The reflex molecular testing of tumor tissue was performed per institutional standard-of-care. TAT from tissue NGS order to result was recorded.

### 2.3. Study Endpoints

The primary objective was to evaluate the difference in TAT between LBx testing and tissue *EGFR* testing. TAT for tissue NGS was defined as the time interval between tissue biopsy and NGS test result. TAT for LBx was defined as the time interval between blood sampling and PCR result. Secondary objectives included the sensitivity and specificity of LBx compared to tissue *EGFR* testing as well as the role of LBx *EGFR* testing in patients in whom EGFR testing was not conducted on tissue biopsy for various reasons.

### 2.4. Statistical Analysis

Descriptive statistics were employed to define clinicopathological characteristics at baseline. Sensitivity reflects the ability of a test to correctly identify the disease or mutation, which was calculated using the formula

Sensitivity = True Positives (TP)/True Positives (TP) + False Negatives (FN) × 100. Specificity reflects the ability of a test to correctly identify a patient who does not have the disease or mutation and was calculated with the formula Specificity = True Negatives (TN)/True Negatives (TN) + False Positives (FP) × 100. The Shapiro–Wilk test was used to evaluate the normality of data distributions, such as differences in TAT for plasma EGFR testing versus tissue NGS in pathology. For datasets deviating from normality, comparisons of matched data entries were conducted using the Wilcoxon test. Statistical significance was defined by a two-tailed α < 0.05. Statistical analyses were performed using R version 4.4.0 (R Foundation for Statistical Computing, Vienna, Austria).

## 3. Results

### 3.1. Population Characteristics

Between August 2019 and February 2023, 366 patients were recruited with a median age at diagnosis of 68 years (range, 32–95 years). Most patients (n = 164, 44.8%) were male and had a smoking history (n = 294; 81.9%). The Eastern Cooperative Oncology Group (ECOG) status was ≥2 in 30.3% (n = 111), and 70.5% (n = 258) of patients had stage IV disease at screening. Brain metastases were present in 24.3% (n = 89) of patients. The baseline demographic and disease characteristics are outlined in Table 1.

### 3.2. Tissue Biopsy

**Figure 1:**
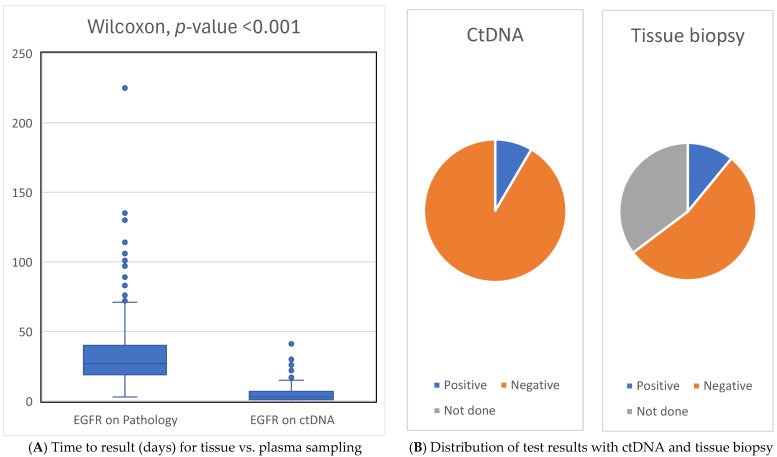
Boxplot of time to result (in days) comparing tissue biopsies (**left**) and plasma ctDNA testing. *Y*-axis = number of days from EGFR test to result (**right**; (**A**)). Distribution of test results with ctDNA and tissue biopsy (**B**).

In total, 356 patients underwent tissue biopsy after ctDNA blood draw, of whom 237 had *EGFR* testing. Among the 129 patients who did not undergo EGFR testing via tissue biopsy, the reasons were distributed as follows: 16 patients (12.4%) had benign histology, 20 (15.5%) had early-stage disease, 20 (15.5%) had insufficient or inadequate tissue for molecular testing, 14 (10.9%) had either small-cell lung cancer (SCLC) or were heavy smokers with squamous cell carcinoma of the lung, 14 (10.9%) had non-lung primary tumors, 13 (10.1%) were either unfit for or declined tissue biopsy, and in 29 cases (22.5%), the reason for not performing molecular testing was unspecified (Figure 1). The median time from tissue biopsy to pathology result was 10 days (3–72 days).

### 3.3. Difference in TAT Between LBx and Tissue EGFR Testing

Among the patients who underwent molecular testing (either LBx or tissue NGS), *EGFR* mutations were detected in 31 (8.5%) and 40 (10.9%) of cases by ctDNA and tissue biopsy, respectively. *EGFR* exon 19 deletion was the most frequent mutation, which was detected in 59% of patients. The median TAT for plasma testing from blood draw to result was 3 days (0–49 days) compared to 26 days (3–225 days) with tissue NGS, which was measured from the tissue test request to reporting (*p*-value < 0.001).

### 3.4. Sensitivity and Specificity Analyses

ctDNA demonstrated 100% specificity and 65% sensitivity for EGFR status. The positive predictive value was 100%, and the negative predictive value was 95%. In addition, seven of the patients who did not have EGFR testing on tissue biopsy because of inadequate or insufficient tissue tested positive for EGFR mutation on LBx.

Of the patients with *EGFR* mutation and cerebral metastasis, only 22.2% of patients underwent brain radiotherapy in the ctDNA-positive group, whereas this number increased to 37.5% in the tissue NGS-positive group.

**Figure 2:**
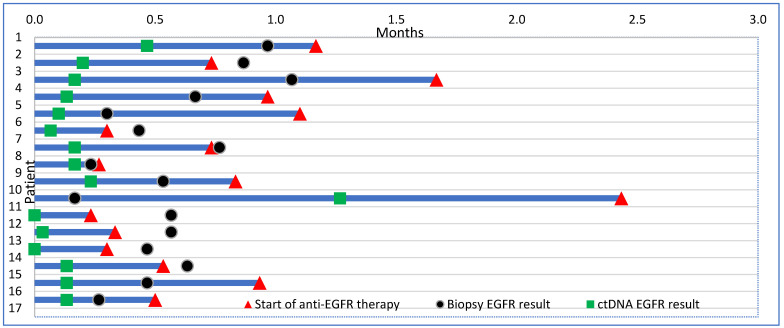
Swimmer’s plot showing time of start of first-line anti-EGFR tyrosine kinase inhibitor (TKI) in relationship to time of ctDNA and tissue NGS result. *X*-axis = months, *Y*-axis = patients.

Data concerning anti-EGFR TKI treatment initiation were available for 16 patients. Among these 16 patients, it is important to highlight that seven (43.7%) started TKI based on ctDNA result and before tissue biopsy (Figure 2).

## 4. Discussion

In our pursuit of enhancing diagnostic precision for aNSCLC, the integration of ctDNA molecular testing emerges as a transformative approach. Our prospective multicenter study underscored the urgency and significance of implementing early ctDNA testing in suspected cases of aNSCLC, aiming to ensure prompt and comprehensive diagnostic evaluations for all patients.

In our study, we presented compelling evidence regarding the potential of ctDNA testing particularly in early detection of *EGFR* mutations upon suspicion of aNSCLC. Compared to traditional tissue biopsy testing, ctDNA yielded results significantly faster with a TAT of 3 days versus 26 days (*p* < 0.001). While acknowledging that ctDNA does not supplant molecular testing performed on tissue biopsy, it served as a crucial tool for the early detection of *EGFR* mutations in a substantial proportion (8.47%) of cases. This timely detection allows for informed treatment decisions, potentially averting unnecessary interventions such as brain radiotherapy or the initiation of chemotherapy. In addition, *EGFR* mutation was detected on ctDNA in seven patients who did not receive EGFR testing on tissue biopsy due to insufficient or inadequate tissue sampling. Thus, our results advocate for the utility of *EGFR* ctDNA testing upon suspicion of aNSCLC, facilitating timely and informed treatment strategies.

Comprehensive genomic profiling remains imperative for patients with aNSCLC, yet persistent clinical practice gaps pose pressing challenges. Aggarwal et al. underscored the profound impact of pre-therapy genomic profiling on OS rates, emphasizing the critical need for timely molecular results [26]. However, Sadik et al. shed light on the disconcerting reality that 50% of patients were excluded from precision oncology pathways due to various obstacles in obtaining biomarker test results, including tissue scarcity, inadequate reflex testing, prolonged turnaround times, and financial barriers [27]. This was reflected in our study with 20 (5.4%) and 29 (7.9%) patients not benefiting from *EGFR* testing due to insufficient or inadequate tissue and unknown reasons, respectively.

A previous study conducted in our academic center revealed a time to detection of *EGFR* mutation from clinical suspicion of 78.7 days and 48.4 days, respectively, in the time periods of 2014–2016 and 2017–2019. Overall, 30% of patients had started chemotherapy as NGS results were not available, and it was associated with worse overall prognosis [28]. Although we could see an improvement of the time to detection of *EGFR* mutation from clinical suspicion in our study, at 26 days, it remained significantly longer compared to LBx NGS testing at 3 days.

Recent studies, such as the French study Libellule and the Canadian study Accelerate, underscored the clinical utility of incorporating plasma ctDNA testing upon radiologic suspicion of NSCLC rather than waiting for a tissue pathology report. These trials reaffirmed the pivotal role of ctDNA testing in expediting treatment initiation, thus advocating for its widespread adoption to ensure timely and comprehensive diagnostic evaluation for all patients with aNSCLC [29,30]. In the Accelerate study, the median TAT for LBx was 7 days versus 23 days for tissue NGS, which mirrors the findings of our study. LBx based on radiologic evidence of lung cancer led to significant reduction in the delay from referral to start of treatment (39 days vs. 62 days). Libellule’s study found that upfront Lbx biomarker testing reduced the time to initiation of treatment by 9.7 days in patients who received systemic treatment and 16.4 days in patients who had a targetable mutation and were started on TKI.

In addition, the retrospective study by Russo et al. found that delay from diagnosis to therapy was significantly reduced in patients who had LBx testing prediagnosis compared to patients who had it postdiagnosis (21 vs. 35 days) [31]. Similarly, a prospective study conducted at the University of Pennsylvania found that plasma-based NGS conducted at the time of biopsy on the basis of imaging results allowed a reduction in time to treatment compared to reflex tissue NGS (12 vs. 20 days) [32].

Despite representing a prospective, multicenter effort providing meaningful data in favor of early testing of patients suspected of having aNSCLC, our study has limitations. First, in a resource-limited healthcare setting, LBx testing represents a relatively expensive diagnostic method considering the low overall prevalence of *EGFR* activating events in the aNSCLC population. Performing ctDNA based on imaging suspicious for lung cancer has proven to be time effective, but efforts still need to be made to optimize its cost-effectiveness. Adequate patient selection strategies remain to be explored. In our study, 310 (84.7%) patients were eventually diagnosed with aNSCLC; however, 56 (15.3%) were ultimately considered inadequate candidates for NGS on tissue biopsy due to early-stage disease or tissue histology consistent with SCLC, non-pulmonary malignancies, or benign lesions. In a state-funded medical system, cost effectiveness is a major factor in adopting a diagnostic study. Cost-effectiveness studies could be performed taking into consideration reduction in hospitalization costs due to chemotherapy side effects as well as reduction in unnecessary procedures such as repeat biopsy and brain radiation therapy. Second, our study only tested for EGFRm, as it remains the most frequent actionable genomic alteration in lung cancer. Ideally, our aim would be to implement ctDNA complete genomic profiling to our routine testing. That would allow the detection of more ESCAT tier 1 actionable mutations, thus serving a bigger proportion of patients.

## 5. Conclusions

In conclusion, our multicenter Canadian study at the provincial level demonstrates the clinical significance of upfront ctDNA *EGFR* testing in aNSCLC based on radiologic suspicion. We hope that our findings will pave the way for broader access to ctDNA.

## Figures and Tables

**Table 1:** Baseline demographics and clinical characteristics for included patients.

Characteristic	N = 366
Age at diagnosis, years, median (IQR)	69 (32–95)
Sex (male-female, %)	45–55
Referring center, No. (%)	
CHUM	309 (84%)
Other centers	57 (16%)
Smoking Habits, No. (%)	
Never	62 (18%)
Former	112 (31%)
Current	183 (51%)
ECOG, No. (%)	
0	84 (22.9%)
1	151 (41.25%)
2	58 (15.84%)
3+	53 (14.48%)
Unknown	20 (5.46%)
Histology, No. (%)	
Adenocarcinoma	247 (67.5%)
Squamous cell	36 (9.8%)
Other NSCLC	27 (7.38%)
SCLC	12 (3.3%)
Other malignancies	14 (3.83%)
Benign	23 (6.28%)
No tissue diagnosis	7 (1.9%)
Stage at diagnosis, No. (%)	
I	11 (3%)
II	20 (5.46%)
III	77 (21%)
IV	258 (70.5%)
CNS metastasis, No. (%)	
No	259 (70.7%)
Yes	89 (24.3%)
Unknown	18 (5%)
Positive *EGFR* by ctDNA	31 (8.47%)
Positive *EGFR* by tissue biopsy	40 (10.9%)

Abbreviations: CHUM = Centre hospitalier Université de Montreal; ECOG = Eastern Cooperative Oncology Group; IQR = interquartile range; NSCLC = non-small cell lung cancer; SCLC = small cell lung cancer; CNS = central nervous system metastasis.

## Data Availability

The data presented in this study are available on request from the corresponding author.

## References

[1] Siegel R.L., Miller K.D., Wagle N.S., Jemal A. (2023). Cancer statistics, 2023. CA Cancer J. Clin..

[2] Mok T.S., Wu Y.-L., Thongprasert S., Yang C.-H., Chu D.-T., Saijo N., Sunpaweravong P., Han B., Margono B., Ichinose Y. (2009). Gefitinib or carboplatin-paclitaxel in pulmonary adenocarcinoma. N. Engl. J. Med..

[3] Ferrara M.G., Di Noia V., D’Argento E., Vita E., Damiano P., Cannella A., Ribelli M., Pilotto S., Milella M., Tortora G. (2020). Oncogene-Addicted Non-Small-Cell Lung Cancer: Treatment Opportunities and Future Perspectives. Cancers.

[4] Singh N., Temin S., Baker S., Blanchard E., Brahmer J.R., Celano P., Duma N., Ellis P.M., Elkins I.B., Haddad R.Y. (2022). Therapy for Stage IV Non–Small-Cell Lung Cancer with Driver Alterations: ASCO Living Guideline. J. Clin. Oncol..

[5] O’Sullivan D.E., Jarada T.N., Yusuf A., Hu L., Xun Y., Gogna P., Brenner D.R., Abbie E., Rose J.B., Eaton K. (2022). Prevalence, Treatment Patterns, and Outcomes of Individuals with EGFR Positive Metastatic Non-Small Cell Lung Cancer in a Canadian Real-World Setting: A Comparison of Exon 19 Deletion, L858R, and Exon 20 Insertion EGFR Mutation Carriers. Curr. Oncol..

[6] Melosky B., Kambartel K., Häntschel M., Bennetts M., Nickens D.J., Brinkmann J., Kayser A., Moran M., Cappuzzo F. (2022). Worldwide Prevalence of Epidermal Growth Factor Receptor Mutations in Non-Small Cell Lung Cancer: A Meta-Analysis. Mol. Diagn. Ther..

[7] Kobayashi S., Boggon T.J., Dayaram T., Jänne P.A., Kocher O., Meyerson M., Johnson B.E., Eck M.J., Tenen D.G., Halmos B. (2005). EGFR Mutation and Resistance of Non–Small-Cell Lung Cancer to Gefitinib. N. Engl. J. Med..

[8] Paez J.G., Jänne P.A., Lee J.C., Tracy S., Greulich H., Gabriel S., Herman P., Kaye F.J., Lindeman N., Boggon T.J. (2004). EGFR mutations in lung cancer: Correlation with clinical response to gefitinib therapy. Science.

[9] Bruno D.S., Hess L.M., Li X., Su E.W., Zhu Y.E., Patel M. (2021). Racial disparities in biomarker testing and clinical trial enrollment in non-small cell lung cancer (NSCLC). J. Clin. Oncol..

[10] Robert N.J., Nwokeji E.D., Espirito J.L., Chen L., Karhade M., Evangelist M.C., Spira A.I., Neubauer M.A., Bullock S.A., Coleman R.L. (2021). Biomarker tissue journey among patients (pts) with untreated metastatic non-small cell lung cancer (mNSCLC) in the U.S. Oncology Network community practices. J. Clin. Oncol..

[11] Yip S., Christofides A., Banerji S., Downes M.R., Izevbaye I., Lo B., MacMillan A., McCuaig J., Stockley T., Yousef G.M. (2019). A Canadian Guideline on the Use of Next-Generation Sequencing in Oncology. Curr. Oncol..

[12] Drilon A., Wang L., Arcila M.E., Balasubramanian S., Greenbowe J.R., Ross J.S., Stephens P., Lipson D., Miller V.A., Kris M.G. (2015). Broad, Hybrid Capture–Based Next-Generation Sequencing Identifies Actionable Genomic Alterations in Lung Adenocarcinomas Otherwise Negative for Such Alterations by Other Genomic Testing Approaches. Clin. Cancer Res..

[13] Johnson D.B., Dahlman K.H., Knol J., Gilbert J., Puzanov I., Means-Powell J., Balko J.M., Lovly C.M., Murphy B.A., Goff L.W. (2014). Enabling a Genetically Informed Approach to Cancer Medicine: A Retrospective Evaluation of the Impact of Comprehensive Tumor Profiling Using a Targeted Next-Generation Sequencing Panel. Oncologist.

[14] Kopetz S., Mills Shaw K.R., Lee J.J., Zhang J., Litzenburger B., Holla V., Kinyua W., Broaddus E., Daniels M.S., Meric-Bernstam F. (2019). Use of a Targeted Exome Next-Generation Sequencing Panel Offers Therapeutic Opportunity and Clinical Benefit in a Subset of Patients With Advanced Cancers. JCO Precis. Oncol..

[15] Sheffield B.S., Eaton K., Emond B., Lafeuille M.-H., Hilts A., Lefebvre P., Morrison L., Stevens A.L., Ewara E.M., Cheema P. (2023). Cost Savings of Expedited Care with Upfront Next-Generation Sequencing Testing versus Single-Gene Testing among Patients with Metastatic Non-Small Cell Lung Cancer Based on Current Canadian Practices. Curr. Oncol..

[16] Ali A., Singh G., Camilleri-Broet S., Cohen V., Spicer J., Wang H., Chong G., Munazzit M., Rousseau C., Sateren W. (2018). P2.01-04 Reducing Time to Molecular Diagnosis for Advanced NSCLC in the Context of a Reference Testing Center. J. Thorac. Oncol..

[17] Ettinger D.S., Wood D.E., Aisner D.L., Akerley W., Bauman J.R., Bharat A., Bruno D.S., Chang J.Y., Chirieac L.R., D’Amico T.A. (2022). Non-Small Cell Lung Cancer, Version 3.2022, NCCN Clinical Practice Guidelines in Oncology. J. Natl. Compr. Cancer Netw. JNCCN.

[18] Scott J.A., Lennerz J., Johnson M.L., Gordan L.N., Dumanois R.H., Quagliata L., Ritterhouse L.L., Cappuzzo F., Wang B., Xue M. (2024). Compromised Outcomes in Stage IV Non–Small-Cell Lung Cancer with Actionable Mutations Initially Treated Without Tyrosine Kinase Inhibitors: A Retrospective Analysis of Real-World Data. JCO Oncol. Pract..

[19] Pantel K., Alix-Panabières C. (2010). Circulating tumour cells in cancer patients: Challenges and perspectives. Trends Mol. Med..

[20] Weber B., Meldgaard P., Hager H., Wu L., Wei W., Tsai J., Khalil A., Nexo E., Sorensen B.S. (2014). Detection of EGFR mutations in plasma and biopsies from non-small cell lung cancer patients by allele-specific PCR assays. BMC Cancer.

[21] Donaldson J., Park B.H. (2018). Circulating Tumor DNA: Measurement and Clinical Utility. Annu. Rev. Med..

[22] Wan J.C.M., Massie C., Garcia-Corbacho J., Mouliere F., Brenton J.D., Caldas C., Pacey S., Baird R., Rosenfeld N. (2017). Liquid biopsies come of age: Towards implementation of circulating tumour DNA. Nat. Rev. Cancer.

[23] Luis A., Diaz J., Bardelli A. (2014). Liquid Biopsies: Genotyping Circulating Tumor DNA. J. Clin. Oncol..

[24] Jee J., Lebow E.S., Yeh R., Das J.P., Namakydoust A., Paik P.K., Chaft J.E., Jayakumaran G., Rose Brannon A., Benayed R. (2022). Overall survival with circulating tumor DNA-guided therapy in advanced non-small-cell lung cancer. Nat. Med..

[25] Health C for D and R (2023). List of Cleared or Approved Companion Diagnostic Devices (In Vitro and Imaging Tools). FDA [Internet]. https://www.fda.gov/medical-devices/in-vitro-diagnostics/list-cleared-or-approved-companion-diagnostic-devices-in-vitro-and-imaging-tools.

[26] Aggarwal C., Marmarelis M.E., Hwang W.-T., Scholes D.G., McWilliams T., Singh A.P., Sun L., Kosteva J.A., Costello M.R., Cohen R.B. (2022). Association of comprehensive molecular genotyping and overall survival in patients with advanced non-squamous non-small cell lung cancer. J. Clin. Oncol..

[27] Sadik H., Pritchard D., Keeling D.-M., Policht F., Riccelli P., Stone G., Finkel K., Schreier J., Munksted S. (2022). Impact of Clinical Practice Gaps on the Implementation of Personalized Medicine in Advanced Non-Small-Cell Lung Cancer. JCO Precis. Oncol..

[28] Blanc-Durand F., Florescu M., Tehfe M., Routy B., Alameddine R., Tran-Thanh D., Blais N. (2021). Improvement of EGFR Testing over the Last Decade and Impact of Delaying TKI Initiation. Curr. Oncol..

[29] Swalduz A., Curcio H., Ambasager B., Moel G.L., Dot J.-M., Duruisseaux M., Fournel P., Odier L., Demolombe S., Byzieux A. (2024). LIBELULE: A randomized phase III study to evaluate the clinical relevance of early liquid biopsy in patients with suspicious metastatic lung cancer. J. Thorac. Oncol..

[30] García-Pardo M., Czarnecka-Kujawa K., Law J.H., Salvarrey A.M., Fernandes R., Fan Z.J., Waddell T.K., Yasufuku K., Liu G., Donahoe L.L. (2023). Association of Circulating Tumor DNA Testing Before Tissue Diagnosis with Time to Treatment Among Patients with Suspected Advanced Lung Cancer: The ACCELERATE Nonrandomized Clinical Trial. JAMA Netw. Open.

[31] Russo A., Lee J.K., Pasquina L.W., Del Re M., Dilks H.H., Murugesan K., Madison R.W., Lee Y., Schrock A.B., Comment L. (2024). Liquid Biopsy of Lung Cancer Before Pathological Diagnosis Is Associated with Shorter Time to Treatment. JCO Precis. Oncol..

[32] Thompson J.C., Aggarwal C., Wong J., Nimgaonkar V., Hwang W.-T., Andronov M., Dibardino D.M., Hutchinson C.T., Ma K.C., Lanfranco A. (2022). Plasma Genotyping at the Time of Diagnostic Tissue Biopsy Decreases Time-to-Treatment in Patients with Advanced NSCLC—Results From a Prospective Pilot Study. JTO Clin. Res. Rep..

